# Role of pronator release in revision carpal tunnel surgery

**DOI:** 10.1051/sicotj/2016006

**Published:** 2016-03-16

**Authors:** Pobe Luangjarmekorn, Tsu Min Tsai, Sittisak Honsawek, Pravit Kitidumrongsook

**Affiliations:** 1 Department of Orthopaedics, Faculty of Medicine, Chulalongkorn University 1873 Rama 4 Road 10330 Pathumwan, Bangkok Thailand; 2 Christian M Kleinert Institute for Hand and Microsurgery 225 Abraham Flexner Way, Suite 850 40202 Louisville KY USA

**Keywords:** Pronator syndrome, Pronator release, Revision, Carpal tunnel, Decompression

## Abstract

*Introduction*: The purpose of this study was to compare the result of treatment of patients with failed primary carpal tunnel surgery who suspected pronator teres syndrome (PTS) by performing revision carpal tunnel release (CTR) with pronator teres release (PTR) and revision CTR alone.

*Methods*: Retrospective chart review in patients who required revision CTR and suspected PTS. Group 1, treated by redo CTR with PTR and group 2, treated by redo CTR alone. Intraoperative findings, pre and postoperative numbness (2-PD), pain (VAS score), and grip strength were studied.

*Results*: There were 17 patients (20 wrists) in group 1 and 5 patients (5 wrists) in group 2. Patients in group 1 showed more chance of fully recovery of numbness and pain than group 2 (60% vs. 0%, *p* < 0.05 and 55.0% vs. 0%, *p* < 0.05, respectively). Mean grip strength was increased 16.0% in group 1 and increase 11.7% in group 2. Most common pathology at the elbow were deep head of pronator teres 90% (18/20 elbows) and lacertus fibrosus 50% (10/20 elbows). The most common finding at carpal tunnel was the reformed transverse carpal ligaments (80%, 20/25 wrists) and scar adhesion around the median nerve (40%, 10/25 wrists).

*Discussion*: Intraoperative findings from our study confirmed that there were pathology in both carpal tunnel and pronator area in failed primary CTR with suspected PTS. Our study showed that combined PTR with revision CTR provided higher chance of completely recovery from numbness and pain more than redo CTR alone.

## Introduction

Carpal tunnel release (CTR) is one of the most common procedures with a high success rate. However, 2.0–25.0% of patients remained persistently symptomatic or developed recurrent symptoms after surgery [1, 2]. Approximately 12.0% of these cases needed revision procedures. The outcomes of revision CTR were disappointing, 40.0% reported unfavorable results and up to 95.0% had persistent symptoms. The most common findings in revision CTR are an incomplete section of flexor retinaculum (54.0–62.0%) and scar tethering (23.0–35.0%) [3–5]. Nevertheless, the other possible causes of failed primary CTR include proximal compression from pronator compression, thoracic outlet compression, or cervical spine problems.

Pronator teres syndrome (PTS), first described in 1951 by Seyffarth [6], is another cause of compressive neuropathy of the median nerve and is usually underestimated by physicians. Diagnosis of PTS is generally made by patients’ symptoms such as volar forearm aching pain, radial three-digit numbness, and aggravated symptoms during repetitive grasping or pronation activities. Various positive pronator aggravation tests and a negative carpal steroid injection can help support the diagnosis of PTS.

Carpal tunnel syndrome (CTS) and PTS can be found together. Our previous study reported 55 patients in whom both lesions were suspected and they underwent simultaneous release of the ipsilateral pronator and carpal tunnel. Benefits of this protocol were limited exposure for two operations, shortened morbidity time and provided satisfactory outcome (86% improved symptom and 65% complete recovery) [7].

The purpose of this study was to compare the outcome between revision CTR with pronator teres release (PTR) and revision CTR alone in the patients who failed primary CTR and suspected PTS.

## Materials and methods

A retrospective chart review was conducted in patients who continued to have median nerve symptoms after CTR or developed new symptoms after surgery. All patients were suspected of concomitant PTS from the symptoms of wrist or forearm pain, paresthesia along median nerve distribution, and positive preoperative evaluation in at least one of these three methods.

1. Positive Tinel’s sign at pronator area.
2. Provocative test for PTS aggravated patients’ symptoms by these four maneuvers.
  2.1. Apply pressure at pronator.
  2.2. Active pronation by patient and resisted with examiner’s force.
  2.3. Passive supination of patient’s forearm by the examiner.
  Resisted middle finger superficialis test.
3. Steroid injection at carpal tunnel did not improve symptoms.

Patients were divided into two groups: Group 1 consisted of patients treated by redo CTR with PTR and group 2 included those treated by redo CTR alone. All redo CTRs were done using the two-incision technique. PTR was performed through a separate incisions. All procedures were performed by a single surgeon. Details of surgical procedures were described below.

**Figure 1.**
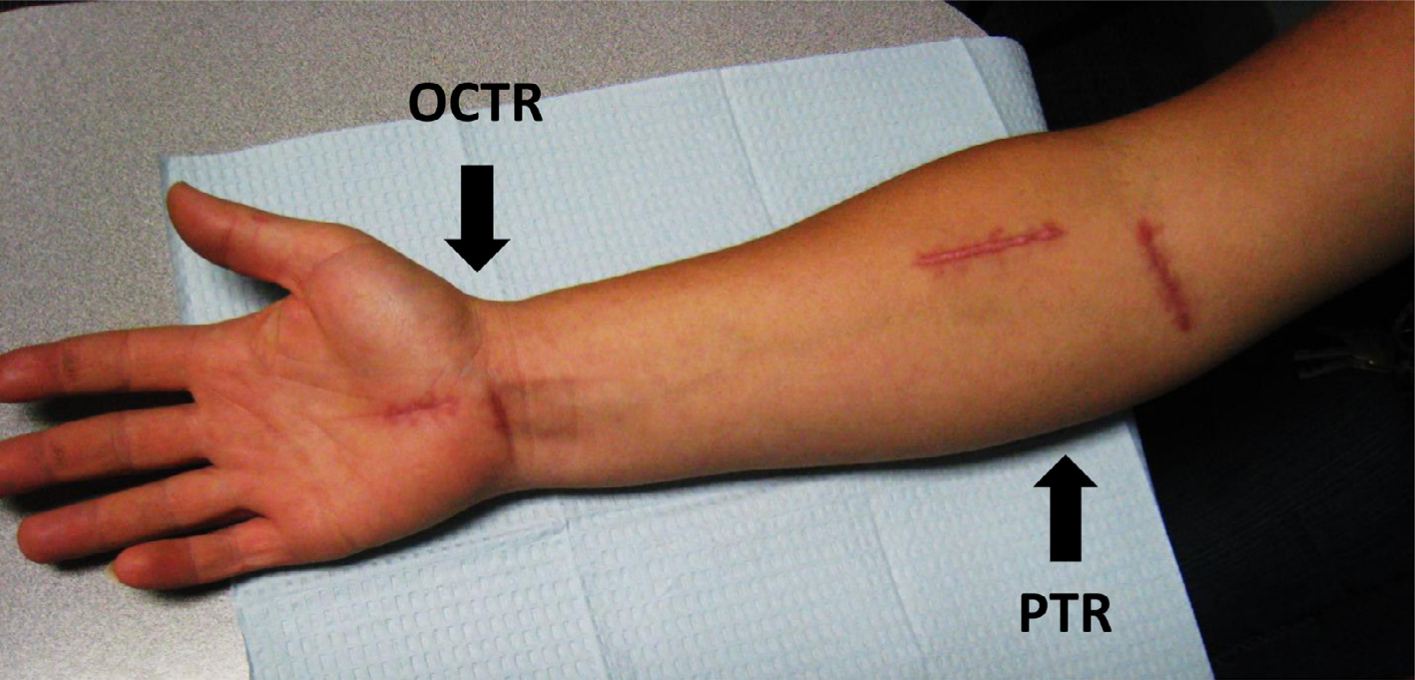
Incisions for pronator teres release (PTR) and opened carpal tunnel release (OCTR).

Two-incision CTR (Figure 1)

- Line was drawn from first webspace to pisiform and vertical line 0.5 cm from thenar crease. First incision extended 1 cm proximally and distally from the initial line. The second incision, 1.0–1.5 cm transverse incision, was done at the distal wrist crease, 1.0 cm ulnar to the scaphoid tubercle. At the palmar incision, the palmar fascia was incised parallel to its fibers exposing the distal part of the transverse carpal ligament (TCL). The reformed TCL or scar above the median nerve was released in a retrograde fashion as far proximal as possible. At the wrist incision, the antebrachial fascia was opened in a proximal-to-distal direction, releasing the TCL, connecting the two limbs of the TCL incisions. Full exploration of the median was visualized via these two incisions [8].

**Figure 2.**
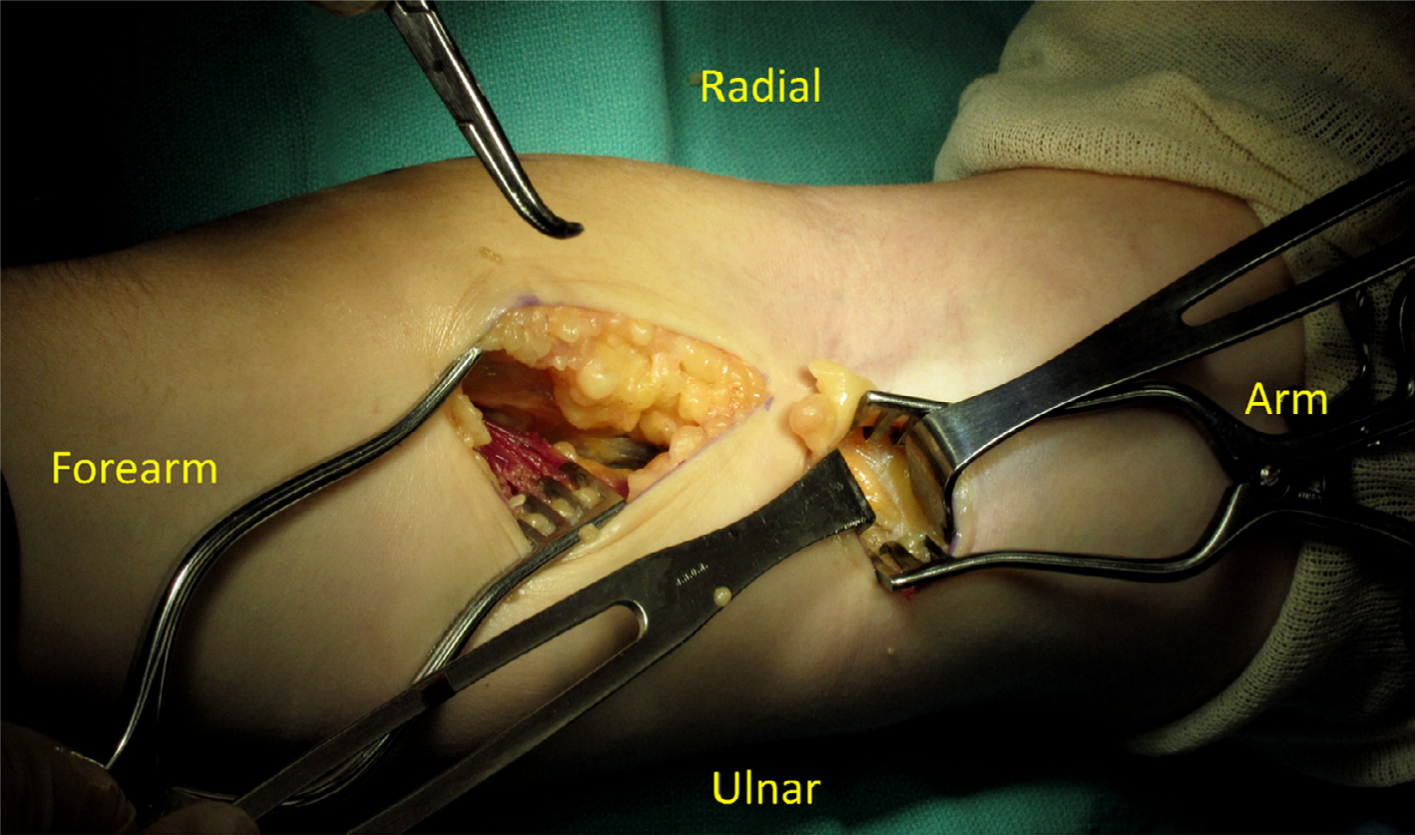
Pronator release by two separated incisions (during operation).

Pronator teres release (Figures 1 and 2)

- 6.0-to-8.0 cm longitudinal incision was performed along the radial edge of flexor-pronator mass (at distal biceps insertion, two-fingerbreadth distal to elbow crease). Preserving the medial antebrachial cutaneous nerve and cubital vein, the fascia of flexor-pronator mass was divided. Then the pronator muscle was retracted to the ulnar side. The median nerve was exposed. Decompression of the nerve was done from proximal-to-distal. The deep head of pronator, FDS arch, and any fibrous band were released by bipolar cautery.
- 3.0-to-4.0 cm second transverse incision was done two-fingerbreadth above elbow crease, ulnar to the biceps muscle. Lacertus fibrosus and brachial sheath were cut. The median nerve was identified. Any fascial bands above the nerve were released from distal-to-proximal.
- Adequacy of decompression was confirmed by insertion of the surgeon’s finger into proximal part, between these two incisions, and distal part along pronator tunnel. If the surgeon palpated any tight structures above the median nerve, it is recommended to connect both incisions or extend the incision proximally to provide better visualization and achieve full decompression of the median nerve.

Intraoperative findings in the carpal tunnel and the pronator region were studied. During follow-up, grip strength was measured by dynamometry and compared between pre- and postoperative values. Pain and numbness were evaluated by using the patient’s interview and pre- and postoperative two-point discrimination (2PD), and visual analog scale (VAS) pain score. Results of treatment were classified into three categories.

Category 1: full recovery

- Full recovery of pain symptom = no postoperative pain that interfered with patient’s activity of daily living (evaluated from patient’s interview) and VAS pain score < 3.
- Full recovery of numbness symptom = no numbness that interfered with patient’s activity of daily living and postoperative 2PD < 10 mm.

Category 2: improved

- Improvement of pain = improvement in postoperative pain (from patient’s interview) and improved postoperative VAS pain score.
- Improvement of numbness = improvement in postoperative numbness and improved 2PD.

Category 3: not improved

- No improvement in pain and numbness (from patient’s interview).
- No improvement in postoperative 2PD.
- No improvement in postoperative VAS pain score.

### Statistical analysis

Statistical analysis was performed using the statistical package for social sciences (SPSS) software, v.16.0 for Windows. Fisher’s exact test was used to compare the difference in pain and numbness. Differences in grip strength were determined using paired *t*-tests. *p* values < 0.05 were considered to be statistically significant.

## Results

There were 22 patients in this study (18 females and four males, total 25 wrists). Mean age was 53.0 years. Mean duration between first CTR and the revision surgery was 4.16 years (2 months–24 years) Preoperative physical examination showed positive for both CTS and PTS signs. Steroid injection at carpal tunnel showed positive effect in 6/16 wrists (37.5%). Electrodiagnostic tests were done in 25 wrists in 22 patients. Twenty-one wrists (84.0%) showed positive for CTS. Only one wrist showed positive for PTS.

There were 17 patients (20 wrists) in group 1 who were treated by PTR with redo CTR (14 unilateral and 3 bilateral diseases). In group 2, there were five patients (all unilateral) who were treated by redo CTR alone. Data in each group are shown in Table 1.

**Table 1.** Demographic data and preoperative evaluation.

	Group 1 PTR + Redo CTR	Group 2 Redo CTR alone
Number of patients	17	5
Number of wrists	20 (3 patients had bilateral)	5
Gender (female/male)	14/3	4/1
Age (years)	52.15 (25–85)	56.0 (32–60)
Physical examination		
Tinel’s at carpal tunnel	15/20	3/5
Phalen’s test	19/20	2/5
Tinel’s at pronator area	15/20	2/5
PTS provocative test	19/20	4/5
Steroid injection at carpal tunnel	8/14 wrists (not improved)	2/2 wrists (not improved)
Electrodiagnostic test		
for CTS	16/20	5/5
for PTS	1/20	0/5

### Intraoperative findings

The intraoperative findings were reviewed from 22 patients. The intraoperative pathology was identified in 20 elbows (from 14 unilateral PTR and 3 bilateral PTR) and 25 wrists (19 unilateral CTR and 3 bilateral CTR).

**Figure 3.**
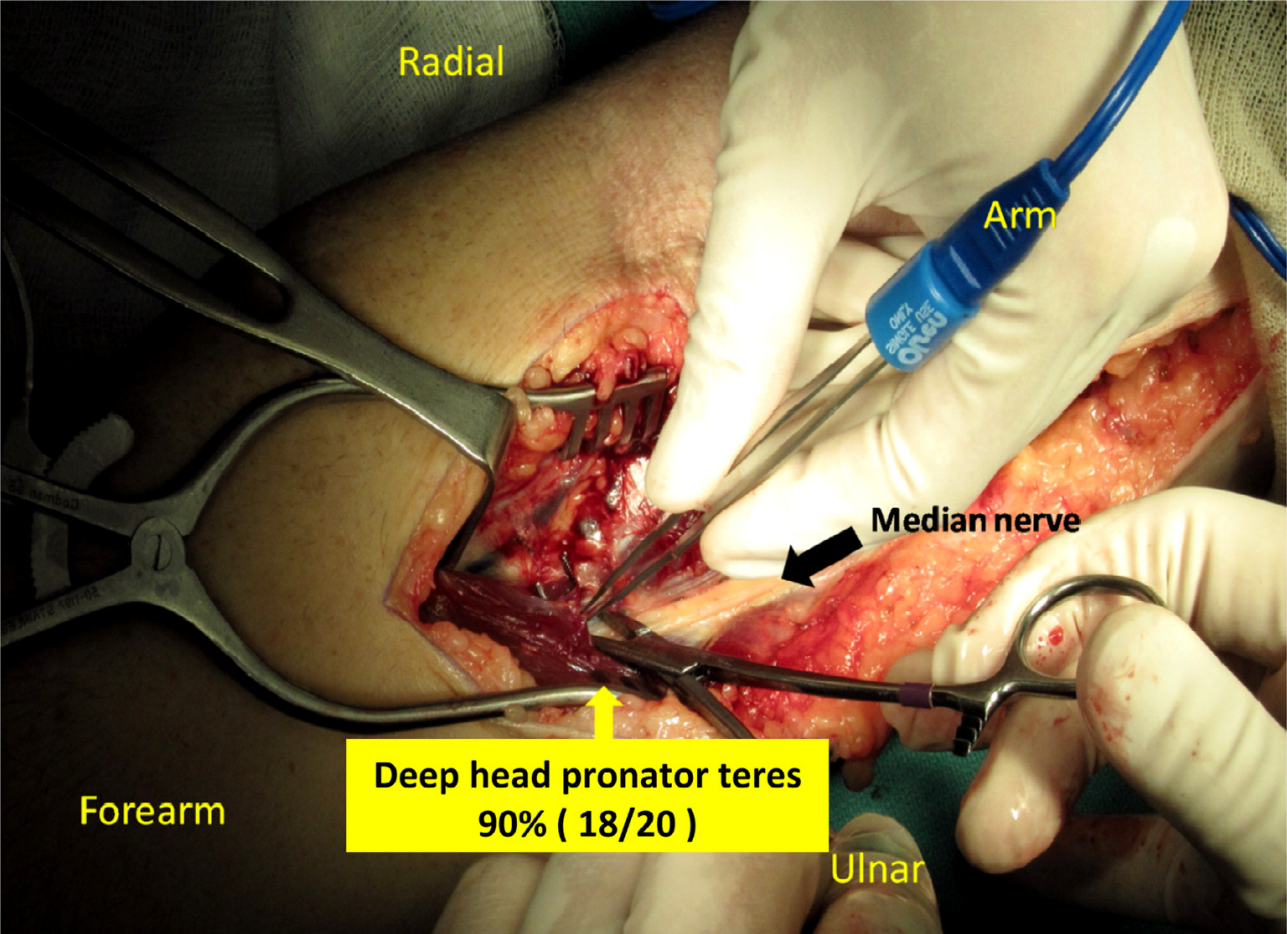
The most common pathologic finding in pronator compression.

The most common structures that caused median nerve compression at the proximal forearm were deep head of pronator teres 90% (18/20 elbows) (Figure 3) and sublimis arch 40% (8/20 elbows). Above the elbow region, the median nerve was usually compressed by lacertus fibrosus 50% (10/20 elbows), Brachial sheath 20% (4/20 elbows), and ligament of Struther 25% (5/20 elbows) as displayed in Table 2.

**Table 2.** Intraoperative findings in PTR and redo CTR*.

Operative findings in redo CTR (total 25 wrists)		Operative findings in PTR (Total 20 elbows)	
Reform TCL**	80% (20/25)	Deep head pronator teres	90% (18/20)
Scar adhesion***	40% (10/25)	Sublimis arch	40% (8/20)
Synovitis	12% (3/25)	Lacertus fibrosus	50% (10/20)
Neuroma	4% (1/25)	Brachial sheath	20% (4/20)
		Ligament of Struther	25% (5/20)

TCL : Transverse carpal ligament.

* Operative findings were reported from 22 patients; 25 wrists (19 unilateral and 3 bilateral CTR), and 20 elbows (14 unilateral and 3 bilateral PTR).

**, *** 4/25 wrists (16%) with reform TCL and 6/25 wrists (24%) with scar adhesion have clear evidence of pressure effect to median nerve including disproportion of median nerve, hour-glass appearance, or tight scar around the median nerve that need external neurolysis.

During redo CTR, the most common finding was the reformed transverse carpal ligaments (80%, 20/25 wrists). But only four wrists of these reformed transverse carpal ligaments (4/25 wrists, 16%) showed clear evidence of pressure effect to median such as disproportion of median nerve or hour-glass appearance. The second most common finding was scar adhesion around the median nerve (40%, 10/25 wrists). Only six wrists with these scar adhesions (6/25 wrists, 24%) showed clear evidence of pressure effect to the median nerve including disproportion of median nerve, hour-glass appearance, or tight scar around the median nerve that needed external neurolysis. Other intraoperative findings in this study were synovitis (3/25 wrists, 12%) and neuroma (1 wrist).

### Postoperative evaluation

From 25 wrists (22 patients) in this study, the symptom of numbness improved after surgery (18/20 wrists in group 1 and 5/5 wrists in group 2). Mean pre- and postoperative 2PD in group 1 were 7.44 mm (ranged 5–10) and 5.94 mm (ranged 4–12). In group 2, pre and postoperative 2PD were 7 mm (ranged 5–10) and 6.33 mm (ranged 6–9). Patients in group 1 showed more chance of full recovery compared with patients in group 2 (12/20 wrists, 60% vs. 0/5 wrist, 0%, *p* < 0.05) as demonstrated in Table 3. For the timing of recovery, 55.0% of wrists in group 1 showed improvement of numbness within two weeks, 70% improved within six weeks, 85% within three months, and 90% within six months. There were two wrists in group 1 (10%) that did not improve after surgery. One case was referred to the spine surgeon because of a cervical spine problem and the other was referred to a thoracic outlet clinic. In group 2, numbness started to improve within two weeks for 40%, while 60% showed improvement within six weeks and all wrists (100%) were improved within three months.

**Table 3.** Surgical outcomes of surgical treatment between two groups.

	Group 1 PTR + Redo CTR (*n* = 20 wrists)	Group 2 Redo CTR alone (*n* = 5 wrists)	*p*
Numbness			
Improved	90% (18/20)	100% (5/5)	1.000
Full recovery	60% (12/20)	0% (0/5)	0.039**
Not improved	10% (2/20)	0% (0/5)	1.000
Pain			
Improved	95% (19/20)	100% (5/5)	1.000
Full recovery	55% (11/20)	0% (0/5)	0.046***
Not improved	5% (1/20)	0% (0/5)	1.000
Grip strength*			
Preoperative	81.2%	63.1%	
Postoperative	97.2%	74.8%	
Improved	+16.0%	+11.7%	0.394

* Grip strength was reported in percentage by comparison with contralateral normal wrist. (Excluded three bilateral diseases in group 1; 6 wrists).

**,*** Statistically significant between group 1 and group 2 (*p* < 0.05).

Pain symptom usually improved after surgery (19/20 wrists in group 1 and 5/5 wrists in group 2). Patients in group 1 showed more chance of full recovery compared with patients in group 2 (11/20 wrists, 55% vs. 0/5 wrists, 0%, *p* < 0.05). Mean pre and postoperative VAS pain score in group 1 were 6.88 (ranged 2–10) and 1.5 (ranged 0–8). For the timing of recovery, in group 1, 55% started to improve within two weeks, 75% within six weeks, 85% within three months, 95% improved in six months, and one patient reported pain and did not improve after surgical intervention. In group 2, pain began to improve within two weeks for 40% of wrists, 60% improved within six weeks, and all wrists (100%) in group 2 had partial pain improvement within three months.

For grip strength evaluation, pre- and postoperative grip strength were measured and compared with contralateral normal side in each patient. After excluding three patients with bilateral diseases (6 wrists), mean pre- and postoperative grip strength in group 1 (14 wrists, unilateral diseases) were 81.2% and 97.2% of contralateral wrists. In group 2 (5 wrists), mean pre and postoperative grip strength were 63.1% and 74.8%. There was a statistically significant improvement in the grip strength of postoperative patients compared with that of preoperative patients (*p* = 0.049 in group 1 and *p* = 0.046 in group 2). Mean grip strength was increased 16.0% in group 1 and increased 11.7% in group 2 (not statistically significant between two groups, *p* = 0.394).

## Discussion

The possible compression sites of median nerve can be at carpal tunnel (51.9–84.5%), pronator area (10.1–37.0%), and supracondylar region (3.7%) [9, 10]. Typically, PTS could be differentiated from CTS by: (1) pain at proximal forearm, (2) numbness at thenar eminence, which was innervated by the palmar cutaneous branch of the median nerve, (3) weakness of extrinsic muscles including pronator quadratus, flexor pollicis longus, and flexor digitorum profundus (FDP) of index and middle finger, (4) lack of nocturnal symptom, and (5) symptom aggravated by pronation activity [11].

Diagnosis of PTS is frequently made by patients’ symptoms and physical examination. Provocative tests would support the diagnosis of PTS and provide the possible pathology of median nerve compression such as “resisted forearm pronation test” (compressed by pronator muscle), “middle finger FDS test” (compressed by FDS arch), and “resisted flexion supination test” (compressed by lacertus fibrosus) [12].

However, there are some overlapping physical signs in pronator compression and carpal tunnel syndrome. Edgell et al. [13] found that 33.3% of CTS had positive Tinel’s sign at the pronator area. In the PTS patients, 48.7% had positive Phalen’s test [14] and 23.1% had positive Tinel’s sign at the carpal tunnel [15].

Steroid injections can be used as diagnostic and therapeutic tools. Symptom improvement after a steroid injection at the pronator muscle supported the diagnosis of PTS. Likewise, a positive effect after carpal tunnel injection can also confirm the diagnosis of CTS. Moreover, a previous study showed that improvement after carpal tunnel injection was a good prognostic factor for higher success rate from carpal tunnel surgery (87.0% vs. 54.0%, *p* < 0.05), compared with the no improvement group [16]. For these reasons, if the patients did not get any improvement from carpal injection, physicians should be aware of less favorable results from carpal tunnel release and look for other possible compression sites other than the carpal tunnel [4, 17].

Electrodiagnostic study for pronator compression showed positive result only in 7.0–31.0% [18], which was very different from compression of median nerve at the carpal tunnel area. In our study, 84% (21/25 wrists) showed positive for CTS but only one case showed positive for PTS. We conclude that the main reason for performing electrodiagnostic study is to exclude other associated nerve conditions such as cervical radiculopathy or other peripheral neuropathy.

Previous literature showed that 50.0–70.0% succeeded with conservative treatments such as limited or modified activity, physical therapy, oral NSAIDs, and immobilization. Surgery might be indicated after a three to six month period of full conservative treatment [14]. Surgical decompression of the median nerve at the elbow could be done by a classic lazy S-incision, modified two longitudinal incisions, single transverse incision, or mini-oblique incision [12, 19, 20]. The overall result of pronator decompression from multiple studies showed 21.6–92.2% complete recovery, 21.0–67.9% partial recovery, and no improvement 9.0–23.0% [6, 12, 13, 21].

Possible causes of failed primary carpal tunnel surgery might result from pathology at the carpal tunnel area itself and other compression sites in the proximal forearm and elbow. The data from this study showed that the physical signs and symptoms of both CTS and PTS in patients who failed primary carpal tunnel surgery were a positive Tinel’s sign at carpal tunnel area 72% (18/25 wrists), positive Phalen’s test 84% (21/25 wrists), positive Tinel’s sign at pronator area 68% (17/25 wrists), and a positive pronator compression test 92% (23/25 wrists). Intraoperative findings in our study also confirmed that there was pathology in both the carpal tunnel and the pronator area. Based on these findings, we advised our patients to have both pronator release and revision carpal tunnel surgery in one setting. By using this protocol, we found that combined PTR with revision CTR provided a higher chance of complete recovery from numbness and pain more than the redo CTR alone (*p* = 0.039 and *p* = 0.046, *p* < 0.05)

However, the pronator teres release was more invasive. This procedure required more complicated surgical skills, deeper dissection, and more invasive anesthetic preparation (brachial block or general anesthesia). In contrast, redo CTR alone was less complicated and could be performed under local anesthesia. Prior to surgery, patients should be advised of the option of less complicated revision carpal tunnel surgery with possible incomplete symptom relief and the more aggressive simultaneous pronator and carpal tunnel decompression with a greater chance of full recovery of their symptoms.

## Conflict of interest

Authors certify that they have no financial conflict of interest in connection with this article.

## References

[1] Abzug JM, Jacoby SM, Osterman AL (2012) Surgical options for recalcitrant carpal tunnel syndrome with perineural fibrosis. Hand 7, 23–29. 23450185 10.1007/s11552-012-9391-7 PMC3280361

[2] Tung TH, Mackinnon SE (2001) Secondary carpal tunnel surgery. Plast Reconstr Surg 107, 1830–1843. 11391209 10.1007/978-3-540-49008-1_40

[3] Jones NF, Ahn HC, Eo S (2012) Revision surgery for persistent and recurrent carpal tunnel syndrome and for failed carpal tunnel release. Plast Reconstr Surg 129, 683–692. 22090245 10.1097/PRS.0b013e3182402c37

[4] Mosier BA, Hughes TB (2013) Recurrent carpal tunnel syndrome. Hand Clin 29, 427–434. 23895723 10.1016/j.hcl.2013.04.011

[5] Stütz N, Gohritz A (2006) Revision surgery after carpal tunnel release – analysis of the pathology in 200 cases during a 2 year period. J Hand Surg Br 31, 68–71. 16257100 10.1016/j.jhsb.2005.09.022

[6] Seyffarth H (1951) Primary myoses in the M. pronator teres as cause of lesion of the N. medianus (the pronator syndrome). Acta Psychiatr Neurol 74, 251. 14902580

[7] Mujadzic M, Papanicolaou G, Young H, Tsai TM (2007) Simultaneous surgical release of ipsilateral pronator teres and carpal tunnel syndromes. Plast Reconstr Surg 119, 2141–2147. 17519713 10.1097/01.prs.0000260703.56453.06

[8] Calleja H, Tsai TM, Kaufman C (2014) Carpal tunnel release using the radial sided approach compared with the two-incision approach. Hand Surg 19(3), 375–380. 25155704 10.1142/S0218810414500300

[9] Bell GE Jr (1956) Compression neuropathy of the median nerve. South Med J 49, 966. 13360461 10.1097/00007611-195609000-00003

[10] Gessini L, Jandolo B, Pietrangeli A (1983) Entrapment neuropathies of the median nerve at and above the elbow. Surg Neurol 19, 112. 6845137 10.1016/0090-3019(83)90405-6

[11] Nigst H, Dick W (1979) Syndromes of compression of the median nerve in the proximal forearm (pronator teres syndrome; anterior interosseous nerve syndrome). Arch OrthopTrauma Surg 93, 307. 10.1007/BF00450231 464765

[12] Zancolli ER III (2012) New mini-invasive decompression for pronator teres syndrome. J Hand Surg Am 37, 1706–1710. 22835590 10.1016/j.jhsa.2012.05.033

[13] Edgell SE, McCabe SJ, Breidenbach WC, LaJoie AS, Abell TD (2003) Predicting the outcome of carpal tunnel release. J Hand Surg Am 28, 255. 12671857 10.1053/jhsu.2003.50031

[14] Hartz CR, Linscheid RL (1981) The pronator teres syndrome: compressive neuropathy of the median nerve. J Bone Joint Surg Am 63, 885. 7240329

[15] Olehnik WK, Manske PR (1994) Median nerve compression in the proximal forearm. J Hand Surg Am 19, 121. 8169356 10.1016/0363-5023(94)90235-6

[16] Rehak D (2001) Pronator syndrome. Clin Sports Med 20, 531. 11494839 10.1016/s0278-5919(05)70267-2

[17] Dahlin LB, Salö M (2010) Carpal tunnel syndrome and treatment of recurrent symptoms. Scand J Plast Reconstr Surg Hand Surg 44, 4–11. 20136467 10.3109/02844310903528697

[18] Rodner CM, Tinsley BA, O’Malley MP (2013) Pronator syndrome and anterior interosseous nerve syndrome. J Am Acad Orthop Surg 21, 268–275. 23637145 10.5435/JAAOS-21-05-268

[19] Gainor BJ (1993) Modified exposure for pronator syndrome decompression: apreliminary experience. Orthopedics 16, 1329–1331. 8108282 10.3928/0147-7447-19931201-07

[20] Tsai TM, Syed A (1994) A transverse skin incision approach for decompression of pronator teres syndrome. J Hand Surg Br 19, 40. 8169477 10.1016/0266-7681(94)90047-7

[21] Johnson RK, Spinner M (1979) Median nerve entrapment syndrome in the proximal forearm. J Hand Surg Am 4, 48. 759503 10.1016/s0363-5023(79)80104-5

